# Return to sport after posterior spinal fusion for adolescent idiopathic scoliosis: what variables actually have an influence? A retrospective study

**DOI:** 10.1007/s43390-022-00535-3

**Published:** 2022-06-20

**Authors:** Alberto Ruffilli, Francesca Barile, Giovanni Viroli, Marco Manzetti, Matteo Traversari, Marco Ialuna, Bartlomiej Dobromir Bulzacki Bogucki, Cesare Faldini

**Affiliations:** Department of Biomedical and Neuromotor Science-DIBINEM, University of Bologna, 1st Orthopaedic and Traumatologic Clinic, IRCCS Istituto Ortopedico Rizzoli, Via Giulio Cesare Pupilli 1, 40136 Bologna, Italy

**Keywords:** Adolescent idiopathic scoliosis, Sport, Return to sport, Posterior spinal fusion

## Abstract

**Purpose:**

To retrospectively evaluate a cohort of athletically active patients who underwent surgery for adolescent idiopathic scoliosis (AIS), and to determine which clinical, surgical and anthropometric variables influenced their return to sport after surgery.

**Methods:**

112 adolescents who underwent high-density posterior fusion for AIS by a single surgeon were analyzed for clinical, surgical and demographic predictors of return to presurgical physical activity levels. Data were retrospectively collected by charts and X-rays analysis and patients interviews.

**Results:**

Preoperative main curve Cobb was 64.4 ± 14.12° and obtained correction was 70.0 ± 12.5%. Included patients played many different sports (Table 4), most of all ballet (44/112, 39.2%), swimming (40/112, 35.7%) and gymnastics (32/112, 28.6%). At an average of 50.3 months follow-up, 76 (67.8%) patients returned to sports (RTS) at an equal or higher level than preoperatively. Younger age, lower Lenke curve type and lower main curve Cobb were significantly associated with RTS. As for RTS timing, patients who returned within the first 6 months were younger, with a higher Lenke and a less severe main curve, a more distal UIV and a more proximal LIV. No complications related to RTS were registered.

**Conclusion:**

In conclusion, patients with adolescent idiopathic scoliosis safely returned to physical activity after surgery. Younger age, higher Lenke type and lower main curve severity predicted a quicker return to sport. However, prospective studies are needed to confirm these findings.

## Introduction

Adolescent idiopathic scoliosis (AIS) affects 2–3% of the population and less than 10% of these patients require surgery [1]. Since it is an asymptomatic disorder, adolescents with AIS are frequently athletically active as age-matched controls [2] and postoperative reduction in sports participation can have detrimental effects on their quality of life [3]; therefore, return to sport (RTS) is an important perioperative concern for the patients and their families.

Nevertheless, current guidelines for postoperative physical activities resumption are mostly derived from expert opinion and a few evidence-based recommendations exist regarding timing of RTS after spinal fusion for AIS [4]. As ability to return to specific sports has not been studied, it is difficult to guide both surgeon and patient with appropriate expectations regarding postoperative RTS.

Aim of this study is to retrospectively evaluate a cohort of athletically active patients who underwent posterior spinal fusion for AIS, and to determine which clinical, surgical and anthropometric variables influenced their return to physical activity.

## Materials and methods

### Study sample

A retrospective review of patients aged 12 to 18 who underwent corrective surgery for adolescent idiopathic scoliosis (AIS) by a single surgeon between 2016 and 2019 was undertaken. Institutional review board approval was obtained before beginning investigation.

Inclusion criteria were: age 12–18 at time of surgery, a diagnosis of AIS, treatment with posterior spinal fusion, a minimum 2-year follow-up, preoperative regular physical activity (other than gym classes at school). Exclusion criteria included: non-idiopathic scoliosis, history of prior spinal surgery, thoracoplasty.

**Fig. 1:**
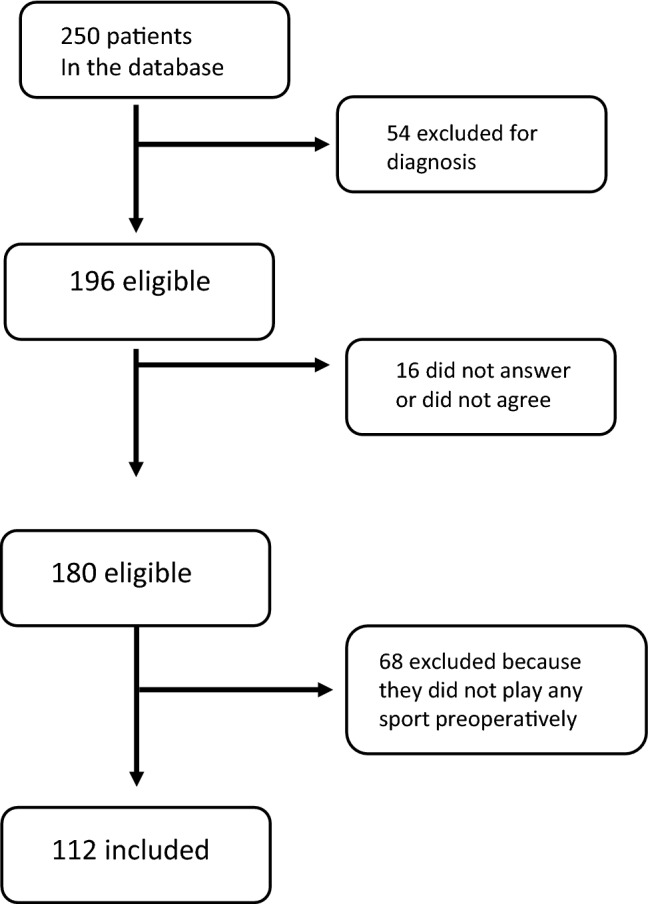
Flowchart of patients’ recruitment

A total of 250 patients were available in the database. Patients with non-idiopathic scoliosis or non-working phone numbers were excluded. 196 patients were contacted by email or phone for participation: 16 did not answer or did not agree, 180 responded and agreed; those who did not play any sport preoperatively (other than gym classes at school) were excluded (68). Return to play at or above the preoperative level (RTS) and the timing for RTS (< 6 months) were assessed (Fig. 1).

### Data collection

Medical records of included patients were reviewed and analyzed. Patients were contacted at different time intervals post-surgery; however, minimum clinical and radiographical follow-up was 2 years. For each patient contact, verbal informed consent to participate was obtained from the patient (or a guardian for < 18 years); patients were asked a series of questions regarding return to school and participation in physical and athletic activities and level of competition, as well as any reasons for any change in level of participation. Responses were confirmed with parent interviews. The Hospital for Special Surgery Pediatric Functional Activity Brief Scale (HSS Pedi-FABS, Table 1) [5], which is routinely administered to all AIS patients before surgery in our institution, and scoliosis research society-22 items (srs-22) questionnaire [6] were then administered. After all responses were collected, reasons for decline in level of participation were listed into categories: back pain, loss of flexibility, deconditioning, loss of desire, scheduling conflicts.

**Table 1:** The HSS Pedi-FABS

	Less than 1 time per month	1 time per month	1 time per week	2–3 times per week	More than 4 times per week
Running: running while playing a sport or jogging					
Cutting: quickly changing directions while running					
Decelerating: coming to a quick stop while running					
Pivoting: turning your body with your foot planted					
Duration: perform athletic activity for as long as you would like without stopping					
Endurance: perform athletic activity for one whole hour without stopping					
Competition: do you participate in organized competitive sports or physical activities?	No (or gym class only)	Yes, but without an official or judge	Yes, with an official or judge	Yes, at a national or professional level	
Supervision: do you participate in supervised (coach, trainer, instructor) sports practice or activities (other than gym class)?	No	Yes, 1–2 times per week	Yes, 3–4 times per week	Yes, 5 or more times per week	

Scoring is performed by adding points from each question for total possible score range from 0 to 30 points. For questions regarding running, cutting, decelerating, pivoting, duration and endurance, each question is worth 0, 1, 2, 3, 4 points. For the questions about competition and supervision, each question is worth 0, 1, 2 or 3 points

Data about operative time, blood loss, fusion levels, instrumentation used, length of stay, intra and postoperative complications were collected. Coronal Cobb’s angle of each curve, % of correction, thoracic kyphosis (TK) and lumbar lordosis (LL) angles were measured on pre and postoperative full length standing radiographs.

### Patients’ characteristics

112 patients (24 males, 88 females) were included, with a follow-up of 50.3 ± 13.9 months. The average age at surgery was 15.6 ± 3.3 years. Patients’ characteristics are summarized in Table 2.

**Table 2:** Patients’ characteristics

Total patients (*n*)	112
Average age (years)	15.6 ± 3.3
Average height (m)	1.6 ± 0.1
Average weight (kg)	57.2 ± 15.5
BMI	20.9 ± 4.3
Curve type—*n* patients	Lenke 1–56
	Lenke 2–12
	Lenke 3–16
	Lenke 4–0
	Lenke 5–8
	Lenke 6–20
Average follow-up (months)	50.3 ± 13.9

*n *number

Patients were all operated by a single surgeon using the same technique (posterior spinal fusion with high-density pedicle screws). They were all cleared for athletic activity (including progression to contact sports) beginning 3 months post-operatively, if they were pain free and implants and curve correction were radiographically unchanged.

### Statistical analysis

Parametric test was used to compare samples in case of continuous variables, normal distribution and appropriate numerousness. The Shapiro–Wilk test was used to verify normal distribution. The Levene test was used to evaluate homogeneity of the variances. As parametric test, we used two-tailed student *t* test to compare the average of the variables for homoscedastic paired groups and Bonferroni correction was used to correct the experiment-wise error rate**.** As nonparametric test, we used the two-tailed Wilcoxon signed-rank test for paired group. Continuity correction was applied in case of discrete distribution.

Binary logistic regression analysis was used to determine the variables that independently predicted delayed return to sport (using 6 months as a cut-off).

Patients were grouped as follows: (1) those who returned to sport at a lower level or did not return to sport; (2) those who returned at the same level or higher. These two groups were compared using independent-sample *t* tests, and Pearson chi-square test for the surgical and demographic variables. A Cochrane-Armitage trend test was used to determine correlation between Lenke classification and return to play. Finally, a binary logistic regression was performed to determine if proximal or distal level of fusion significantly affected the ability to return to athletics earlier (< 6 months). Patients were grouped by lowest fused vertebra and compared using a one-way analysis of variance tests and Pearson chi-square tests. *P* values < 0.05 were considered to be significant. SPSS 17.0 statistical analysis software (SPSS INC., Chicago, Illinois, USA) was used to perform statistical analysis.

## Results

The most frequent scoliosis type was Lenke 1 (56/112, 50%), followed by 6 (20/112, 17.8%) and 3 (16/112, 14.3%); preoperative Cobb angle of the main curve was 64.4 ± 14.12° (range 43–100) and the obtained correction was 70.0 ± 12.5%, with an average postoperative Cobb of 19.6 ± 10.8°.

Levels fused were 11.9 ± 1.2 (range 9–14), with 23.3 ± 2.5 implanted screws on average. Density was high in all cases: average of 1.9 ± 0.04, range 1.8–2.

No intraoperative complications (neither mechanical nor neurological) were registered. Postoperatively, three patients had an early surgical site infection (SSI), successfully treated by surgical debridement and antibiotic therapy; five patients reported anesthesia of a thoracic dermatome.

All patients were discharged within 9 days (average 6.2 ± 1.5, range 4–9). As for return to school/college, the mean time was 6.2 ± 1.9 weeks (range 4–13). SRS-22 was 3.9 ± 1.2 at last follow-up. Surgical and postoperative data are summarized in Tables 3 and 4.

**Table 3:** Surgical data

Average fused levels	11.9 ± 1.2
Average pedicle screws implanted	23.3 ± 2.5
Screw density	1.9 ± 0.04
Average surgical time (min)	228.5 ± 35.4
Average blood loss (ml)	445.3 ± 127.6
Average length of stay (days)	6.2 ± 1.5
Intraoperative complications (mechanical or neurological)	None

**Table 4:** Postoperative data

	Preoperative	Postoperative
Cobb angle of the main curve (% of correction)	64.4 ± 14.12°	19.6 ± 10.8° (70.0 ± 12.5%)
TK	24.9 ± 11.9°	23.7 ± 6.8°
LL	48.7 ± 12.0°	46.2 ± 10.5°
Postoperative complications		3 SSI 5 cases of anesthesia of a thoracic dermatome

*TK *thoracic kyphosis, *LL *lumbar lordosis, *SSI *surgical site infection

Included patients played many different sports (Table 5), most of all ballet (44/112, 39.2%), swimming (40/112, 35.7%) and gymnastics (32/112, 28.6%). 23 patients played more than 1 sport.

**Table 5:** Preoperative and postoperative activities

Activity	Preoperative (*n* patients)	Last follow-up (*n* patients)
None	–	15
Swimming	40	44
Ballet	44	4
Gym	32	60
Volleyball	8	0
Cycling	4	4
Tennis	4	0
Horseback riding	4	4
Skating	4	0
Handball	4	4
Running	4	4
Soccer	4	0

Some patients played more than 1 sport

*n *number

Among the 112 included patients, 76 (67.8%) returned to sport at the same level or higher (RTS group), while 36 (32.2%) did not return at all or returned to lower level (NRTS group). When asked for the reason why they did not resume activities or did not achieve the same results, most of NRTS patients reported stiffness (15/36, 40%), while 9 (25%) answered that the pediatrician or the parents suggested not to play anymore or to play at a lower level/frequency (Table 6).

**Table 6:** Reasons why some patients did not return to a regular physical activity after surgery

Reasons for NRTS	No of patients (*n* = 36)
Stiffness	15
Deconditioning	2
Pain	1
Loss of desire	6
Schedule problems	4
Fear of injury	3
Parents or pediatrician suggestion	9

Some patients chose more than 1 reason

The two groups (RTS and NRTS, Table 7) were significantly different in age, scoliosis type (according to Lenke classification) and main curve severity (preoperative Cobb). In particular, RTS patients were younger (14.9 ± 2.8 vs 16.9 ± 3.7, *p* = 0.03) and had a less severe main curve (61.5 ± 14.1 vs 70.4 ± 12.3, *p* = 0.04).

**Table 7:** Influence of the single variables on sports resumption

Variable	RTS (*n* = 76, 67.8%)	NRTS (*n* = 36, 32.2%)	*p* value
Age (years)	14.9 ± 2.8	16.9 ± 3.7	0.03*
F/M	56/20	32/4	0.77
BMI	21.4 ± 4.4	20.0 ± 4.1	0.12
Operative time	225.1 ± 34.3	235.8 ± 36.9	0.17
Intraoperative blood loss	445.1 ± 129.1	445.8 ± 126.3	0.15
Length of stay	6.2 ± 1.7	6.2 ± 1.0	0.92
Follow-up	50.3 ± 13.9	52.4 ± 14.9	0.60
Preoperative Lenke—*n*. patients	Lenke 1–24	Lenke 1–32	< 0.01*
	Lenke 2–12	Lenke 2–0	
	Lenke 3–16	Lenke 3–0	
	Lenke 4–0	Lenke 4–0	
	Lenke 5–8	Lenke 5–0	
	Lenke 6–16	Lenke 6–4	
Preoperative main curve Cobb	61.5 ± 14.1	70.4 ± 12.3	0.04*
Postoperative main curve Cobb	18.7 ± 10.3	21.3 ± 11.7	0.47
Correction (%)	70.3 ± 11.4	69.5 ± 14.6	0.72
Preoperative TK	26.0 ± 12.3	22.7 ± 10.9	0.30
Postoperative TK	21.4 ± 5.4	25.6 ± 7.9	0.13
Preoperative LL	48.8 ± 12.9	48.4 ± 9.2	0.50
Postoperative LL	43.8 ± 9.3	49.6 ± 9.7	0.06
UIV—*n* patients	T3 or proximal-48	T3 or proximal-24	0.72
	T4 or distal-28	T4 or distal-12	
LIV—*n* patients	L3 or proximal-60	L3 or proximal-32	0.26
	L4 or distal-16	L4 or distal-4	
N. of levels fused	11.8 ± 1.5	12.0 ± 0.8	0.54
Preoperative HSS Pedi-FABS	11.3 ± 5.6	13.2 ± 5.0	0.23
Return to school (weeks)	6.1 ± 1.9	6.2 ± 1.9	**0.89**
SRS-22 (total)	4.0 ± 0.4	3.9 ± 0.9	**0.72**

*RTS *return to sport at the same level or higher than preoperatively, *NRTS *non return to sport or return at lower level or frequency than preoperatively, *UIV *Upper Instrumented Vertebra, *LIV *Lower Instrumented Vertebra, * significant, *n *number, *SRS*-22 scoliosis research society questionnaire, item 22

None of the intraoperative and postoperative parameters was found to significantly influence the return to sport. Also, preoperative intensity of physical activity (HSS Pedi-FABS) was found to be non-significant (*p* = 0.23).

All included patients were cleared for athletic activity beginning 3 months post-operatively. Nevertheless, among the 76 RTS patients, only 12 immediately resumed their previous activities at 3 months; 28 returned to sport between 3 and 6 months and 36 returned after more than 6 months.

When stratifying them according to return to sport timing (Group A, RTS ≤ 6 months and group B, RTS > 6 months, Table 8), some differences between the two groups were found. Infact, patients in group A were significantly younger (14.0 ± 2.5 vs 16.5 ± 3.3, *p* = 0.02), had a less severe main curve (56.4 ± 11.1 vs 67.2 ± 14.9, *p* < 0.01), a more distal UIV (*p* = 0.012) and a more proximal LIV (*p* = 0.025). Also curve type was found to be significantly different between the two groups, with a higher Lenke associated to an earlier RTS (*p* < 0.01).

**Table 8:** Influence of the single variables on the timing of sports resumption

Variable	Group A, RTS ≤ 6 months (*n* = 40, 52.6%)	Group B, RTS > 6 months (*n* = 36, 47.4%)	*p* value
Age (years)	14.0 ± 2.5	16.5 ± 3.3	0.02*
F/M	32/8	24/12	0.19
BMI	21.5 ± 4.7	21.3 ± 4.2	0.56
Operative time	227.7 ± 30.5	222.9 ± 38.4	0.60
Intraoperative blood loss	424.7 ± 121.5	467.8 ± 135.1	0.15
Length of stay	6.3 ± 1.8	6.1 ± 1.5	0.63
Follow-up	51.1 ± 13.1	49.3 ± 14.9	0.58
Preoperative Lenke—*n*. patients	Lenke 1–12	Lenke 1–8	< 0.01*
	Lenke 2–0	Lenke 2–12	
	Lenke 3–9	Lenke 3–4	
	Lenke 4–0	Lenke 4–0	
	Lenke 5–4	Lenke 5–4	
	Lenke 6–8	Lenke 6–8	
Preoperative main curve Cobb	56.4 ± 11.1	67.2 ± 14.9	< 0.01*
Postoperative main curve Cobb	17.1 ± 6.7	20.6 ± 13.2	0.15
Correction (%)	69.7 ± 10.1	70.9 ± 12.9	0.65
Preoperative TK	25.2 ± 10.6	26.9 ± 14.1	0.56
Postoperative TK	24.1 ± 6.3	25.6 ± 7.9	0.38
Preoperative LL	48.7 ± 11.7	49.0 ± 14.7	0.65
Postoperative LL	45.4 ± 11.5	49.6 ± 9.7	0.11
UIV—*n*. patients	T3 or proximal-20	T3 or proximal-28	0.012*
	T4 or distal-20	T4 or distal-8	
LIV—*n*. patients	L3 or proximal-36	L3 or proximal-24	0.025*
	L4 or distal-4	L4 or distal-12	
*N* of levels fused	11.7 ± 1.3	12.0 ± 1.6	0.38
Preoperative HSS Pedi-FABS	12.1 ± 4.2	10.4 ± 6.9	0.21
Return to school (weeks)	6.2 ± 1.8	6.1 ± 2.0	**0.89**
SRS-22 (total)	3.9 ± 1.1	4.0 ± 0.9	**0.64**

*RTS *return to sport, *UIV *Upper Instrumented Vertebra, *LIV *Lower Instrumented Vertebra, *n *number, *SRS*-22 scoliosis research society questionnaire, item 22

## Discussion

Adolescents with AIS are typically active and participate in organized athletic activities alongside their unaffected peers, until the time of surgery [2]. There is substantial agreement that, after spinal fusion, they can perform maximal-effort sport movements without injury [7–9] and can produce comparable trunk and lower extremity kinematics as their healthy peers while participating in high intensity activities, such as running and jumping [8, 9]. Nevertheless, there is a paucity of studies that evaluate variables that influence the return to sport (RTS) after surgery [4, 10–12].

According to the presented results, high-density posterior spinal fusion (PSF) for AIS confirmed to be a safe (no major complications) and effective technique (70.0 ± 12.5% correction of the main curve), that allowed patients to return safely to any kind of physical activity within a few months from surgery [13]. Moreover, the vast majority of included patients (67.8%) reached at least the same level as preoperatively (primary outcome of the study), more than half of them (52.6%) within 6 months (secondary outcome of the study).

Lenke classification was found to have a significant influence on both outcomes; more specifically, patients with higher lower Lenke curve types (IV–VI) returned to play earlier (< 6 months) and reached at least the same level as preoperatively (RTS group), whereas those with lower Lenke curve types (I–III) were more likely to return later (> 6 months) and at a lower level (NRTS group). This result was complemented by the significant association found between the Upper Instrumented Vertebra and the timing of RTS; infact, patients fused distally to T4 were more likely to return to sport earlier (< 6 months); as for the lower instrumented vertebra, it did not affect the likelihood of eventually reaching the preoperative activity level (RTS). This is partially in contrast with Fabricant et al. [1] and Lonner et al. [12], who both found a strong inverse correlation between a more distal fusion and the probability of resuming sport at all. However, these two studies were conducted on a very small number of patients (25 and 38, respectively); moreover, in 2013, a survey conducted on high volume surgeons downplayed LIV as a factor for returning to sports [14]. Tarrant et al. [11] al and Sarwahi et al. [15] also did not find any correlation between LIV and RTS.

Considering the severity of the main curve (preoperative Cobb’s angle), it was associated with both outcomes: patients in the NRTS and patients who returned to sport later had a significantly higher main curve Cobb’s angle. Sarwahi et al. [15] reported a similar finding: patients with Cobb > 70° were more likely to return to sports later.

The only parameter that, other than Lenke and Cobb, was found to have an influence on both outcomes was age (older patients were more likely to return to sport later or not to return at all); this result was not reported by other authors and could be related to natural progression through adolescence, parental influence or other social rather than surgical factors [15, 16]. Interestingly, sagittal plane data (preoperative and postoperative TK and LL), were found to have no influence on RTS. These findings cannot be compared to current literature because, to the Authors’ knowledge, none of the existing studies analyzed them.

With regard to sports and activities played, even though the vast majority of included patients returned to activity at or above the preoperative level, some changed sport: almost all the patients who preoperatively played ballet, volleyball and soccer, post-operatively chose gym and swimming. Other authors reported similar results: while swimming, horseback riding and athletics were in general the most popular sports before surgery [1, 16, 17], gym, cycling and swimming were preferred after surgery [17, 18] in particular, a decrease has been reported in cheerleading and gymnastics, activities which require a high level of truncal flexibility [1, 16, 17, 19–21]. As for return to collision sports, conclusions could not be drawn because none of the included patients played any collision sport preoperatively.

The main limitation of the present study is its retrospective design. Moreover, the timing of return to sport and other activities can be influenced by a number of factors that are not possible to evaluate, ranging from parental influence to socioeconomic and psychosocial variables. Therefore, strong evidence on this topic is very difficult to obtain. Another strong limitation is the lack of longitudinal data about this cohort, since the questionnaire was administered only once.

## Conclusion

In conclusion, patients with Adolescent Idiopathic Scoliosis safely returned to physical activity after surgery. Younger age, higher Lenke type and lower main curve severity predicted a quicker return to sport. However, prospective studies are needed to confirm these findings.
